# Genetic Diversity and Collection Structure Studies of Sesame (*Sesamum indicum* L.) Accessions Across Ethiopian Research Centers

**DOI:** 10.3390/genes17030300

**Published:** 2026-02-28

**Authors:** Feyisa Bejiga Gelashe, Arsénio D. Ndeve, Temesgen M. Menamo, Harish Gandhi, Rogério M. Chiulele

**Affiliations:** 1 Department of Crop Production, Faculty of Agronomy and Forestry Engineering, Eduardo Mondlane University, 3453 Avenida Julius Nyerere, Maputo P.O. Box 257, Mozambique; feyisa.bejiga@ju.edu.et (F.B.G.); ndevegod@gmail.com (A.D.N.); chiulelerogerio@gmail.com (R.M.C.); 2 Department of Plant Science and Horticulture, College of Agriculture and Veterinary Medicine, Jimma University, Jimma P.O. Box 307, Ethiopia; 3 Dryland Legumes and Cereals Program, International Maize and Wheat Improvement Center (CIMMYT), ICRAF House, United Nations Avenue, Nairobi P.O. Box 1041-00621, Kenya; harish.gandhi@cgiar.org; 4 Centre of Excellence in Agri-Food Systems and Nutrition (CE-AFSN), Eduardo Mondlane University, 5° Andar, Edificio da Reitoria, Praça 25 de Junho, Maputo P.O. Box 257, Mozambique

**Keywords:** *Sesamum indicum* L., genetic diversity, collection structure, GBS, DArTSeq, SNP markers, cluster analysis, PCA, collection diversity

## Abstract

Background/Objectives: Despite its economic importance, the genome-wide genetic diversity of sesame germplasm conserved in the Ethiopian national ex situ collection, a proposed center of origin, remains inadequately characterized. This study assessed genome-wide genetic diversity and population structure in 188 sesame accessions from six Ethiopian Agricultural Research Centers using DArTSeq-based SNP markers. Methods: After quality filtering, 5163 high-quality markers were retained from the original set of 12,302 SNPs. Mean expected heterozygosity (He = 0.201) exceeded observed heterozygosity (Ho = 0.193), reflecting sesame’s predominantly self-pollinating nature. Results: The SNPs showed a transition/transversion ratio of 1.17:1 and an uneven distribution across 16 linkage groups. STRUCTURE, PCA, DAPC, and neighbor-joining cluster analyses revealed a clear hierarchical population structure with distinct clusters and varying admixture. Accessions from Assosa (AARC) and Bako (BARC) were genetically uniform, whereas Werer (WARC) and Gambella (GaARC) were major diversity reservoirs, exhibiting high heterozygosity and gene diversity. Pairwise FST values ranged from 0.001 to 0.356, and AMOVA indicated that 30–43% of variation occurred among collections and 57–70% within collections, highlighting substantial intra-collection diversity. Conclusions: The findings highlight that specific research centers were identified as key sources of genetic variation for breeding, conservation, and association mapping to enhance the improvement in agronomic and adaptive traits in sesame for the Ethiopian sesame gene pool.

## 1. Introduction

Sesame (*Sesamum indicum* L.) is a self-pollinating crop belonging to the family Pedaliaceae. Its origin and evolution have long been debated [1,2], with proposed centers including India, the Middle East, and sub-Saharan Africa, including Ethiopia [3,4,5]. However, limited archeological evidence and few experimental studies within the *Sesamum* genus make its evolutionary history difficult to resolve [6]. Thus, it is challenging to pinpoint the crop’s precise origin with accuracy due to all these assertions. Cytogenetically, sesame species are grouped into three classes based on chromosome number: diploid *S*. *indicum* and *S*. *alatum* (2n = 2x = 26), diploid *S*. *latifolium* (2n = 2x = 32), and allotetraploid *S*. *radiatum* (2n = 4x = 64) [7]. A total of 36 sesame species have been reported [8], and the presence of indigenous species in Ethiopia, including *S*. *alatum & S*. *indicum* (2n = 26) and *S*. *latifolium* (2n = 32), supports the country as a center of origin for sesame [9].

Sesame is widely cultivated in tropical regions of the world, with major production in Sudan, India, Myanmar, China, Tanzania, Nigeria, Burkina Faso, Chad, the Central African Republic, and Ethiopia [10,11]. It is valued for its high-quality edible oil (~55%) [12,13] and substantial protein content (18–25%) [14]. In sub-Saharan Africa, sesame has transitioned from a marginal crop to a major export commodity, though smallholder farmers predominantly produce it [15]. In Ethiopia, sesame is widely grown in central and northern regions [16] and is the country’s largest oilseed export, valued at USD 307 million, ranking second only to coffee among agricultural exports [17]. Due to its seed color, size, sweet flavor, natural aroma, and production mostly under organic farming systems, Ethiopian sesame seed is highly favored in premium markets [18]. Despite its economic importance and suitability for genetic studies, genetic improvement of sesame has lagged behind that of other major oilseed crops, highlighting the need for more efficient breeding strategies.

Conventional breeding methods have long been used in oilseed crops to develop new genotypes with desirable traits. However, these approaches are slow and labor-intensive, making them insufficient to meet the growing global demand for oilseeds in the face of an increasing population and declining agricultural resources [19]. Consequently, molecular breeding techniques, which utilize molecular markers, have become valuable tools for characterizing and evaluating genetic diversity both between species and among populations. In sesame, understanding and harnessing genetic diversity is essential for enhancing global productivity. Recent studies have explored sesame genetic diversity using DNA-based markers across various global collections [20,21,22,23]. These markers are particularly useful and reliable, as they remain stable under different environmental conditions [24].

Understanding the extent and pattern of genetic diversity within gene pools is essential for plant breeders to develop improved varieties with desirable traits [25]. In sesame, genetic diversity has been widely assessed using morphological, biochemical, and molecular markers worldwide [26,27,28,29,30,31,32,33,34]. However, studies on Ethiopian sesame germplasm remain limited, with only a few reports based on a limited number of accessions [35], and very few molecular marker-based studies on the local sesame collections. Globally, sesame genetic diversity has been examined using various molecular markers, including amplified fragment length polymorphism [36], sequence-related amplified polymorphism [37], inter-simple sequence repeat [34], simple sequence repeat [38], expressed sequence tag [33], and insertion–deletion markers [39].

Recent advances in molecular genetics have led to the emergence of single-nucleotide polymorphism (SNP) markers. SNP markers are precise, cost-effective, and high-throughput tools with several advantages over earlier markers [40,41]. They are abundant, stable, genome-wide, and efficiently assayed using automated genotyping platforms. Among available methods, genotype-by-sequencing (GBS) is currently the most widely used approach for SNP discovery in plants [42], generating robust marker datasets ranging from tens to thousands, compared with earlier SNP arrays [43]. GBS has been widely applied to assess genetic diversity and population structure in many crops, including sesame [44,45]. However, the genetic diversity and population structure of sesame germplasm conserved in Ethiopian institutional collections remain poorly understood at the genome-wide level. Previous studies have been limited by small sample sizes or few molecular markers, leaving gaps in our knowledge of variation among accessions and across research centers, as well as the impact of curation and regeneration practices. This study, therefore, aims to characterize the genome-wide diversity of one-hundred eighty-eight Ethiopian sesame accessions using the GBS protocol DArTSeq-derived SNPs markers, resolve collection diversity and genetic relationships, and inform germplasm management and breeding, including the identification of diverse accessions for conservation and crop improvement.

## 2. Materials and Methods

### 2.1. Plant Materials

This study evaluated 188 genetically diverse sesame accessions obtained from six different Ethiopian Institutes of Agricultural Research (Table S1). Specifically, 21 accessions from Assosa Agricultural Research Center, 28 accessions from Bako Agricultural Research Center, 12 accessions from Gambela Agricultural Research Center, 36 accessions from Gondar Agricultural Research Center, 21 accessions from Pawe Agricultural Research Center, and 70 accessions from Werer Agricultural Research Center were collected. These genetic materials were collected through the centers from various regions across the country, representing a diverse range of agroecologies.

It is important to note that the accessions analyzed in this study represent ex situ germplasm collections rather than natural biological populations. The genetic structure observed herein is a product of both historical evolutionary processes and human-mediated factors. Specifically, the diversity within these research center groupings has been shaped by the following:

- Purposeful Sampling: Institutional priorities for specific traits or geographic regions.
- Management Bottlenecks: Potential loss of rare alleles during repeated cycles of seed increase and regeneration.
- Managed Mating: The use of closed mating systems during germplasm maintenance.
- Germplasm Exchange: The historical movement and sharing of accessions between research centers.

Consequently, the clusters identified via STRUCTURE and DAPC are interpreted as genetic groups reflecting the curation history and provenance of the accessions, rather than independent evolutionary units driven solely by migration–drift–selection dynamics.

### 2.2. Genomic DNA Extraction and Sequencing

**Figure 1:**
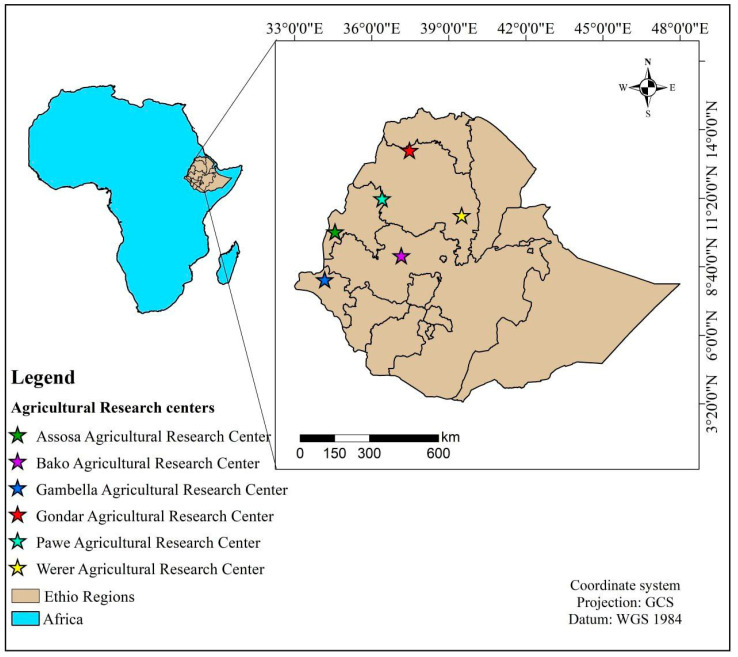
A map showing the collection areas of sesame accessions across Ethiopian research centers.

One-hundred eighty-eight (188) sesame accessions were grown under greenhouse conditions at the Horticulture and Plant Science Department, College of Agriculture and Veterinary Medicine, Jimma University (JUCAVM), Ethiopia, in July 2025 (Figure 1). Four seeds from each accession were planted in a seedling tray and maintained. Samples were collected from fifteen-day-old, fresh, young, and healthy leaf material from the four seedlings for each of the 188 sesame accessions, stored in two 96-well sample collection plates. The leaf tissue samples were stored at −80 °C until lyophilization, and the samples were then dried in a freeze-dryer (Alpha 1-2 LD-plus, Osterode am Harz, Germany) machine. Afterwards, the leaf samples were shipped to SEQART AFRICA at the International Livestock Research Institute (BecA-ILRI) Hub in Nairobi, Kenya, for genotyping.

Genomic DNA was extracted from leaf tissue using the NucleoMag Plant DNA extraction kit, yielding DNA concentrations ranging from 50 to 100 ng/μL. The quality and quantity of the extracted DNA were assessed on 0.8% agarose gels. Libraries were constructed following the DArTSeq complexity reduction protocol [46], which involved digesting genomic DNA with *PstI* and *MseI* enzymes, ligating barcoded and common adapters, and amplifying the adapter-ligated fragments via PCR. Sequencing was performed as single-end reads over 138 cycles on the NovaSeq X platform, following a quality check using GBS as described by [42], employing DArTseq™ technology at the SEQART AFRICA (https://www.seqart.net accessed 12 December 2025) genotyping lab. DArTsoft14, an internal marker scoring pipeline built on algorithms, was used to score DArTseq markers. Two types of DArTseq markers were scored: SilicoDArT and SNP markers. For specific analyses requiring genomic representation in binary form, both marker types were scored as the presence (1) or absence (0) of the corresponding restriction fragment. However, SNP markers were otherwise treated as codominant allelic/genotypic data, and conversion to binary format was strictly limited to analyses where it was methodologically required, and not applied to downstream estimates of genetic diversity and population structure inference. Additionally, to ascertain chromosome positions, both SilicoDArT and SNP markers were aligned to the reference genomes of Chrom_Sesame.

### 2.3. SNP Calling and Data Filtering

The data were filtered after using DArTSeq technology and were analyzed using the R package dartR [47]. Single-nucleotide polymorphism (SNP) markers with less than 10% missing data, minor allele frequency (MAF) greater than 5%, or unknown position were removed for further analysis using a new version of R 4.5.2 software. The SNP markers generated were aligned to the sesame reference genome Zhongzhi No. 13 [13] to determine their positions along the 16 sesame chromosomes. The following criteria were used to filter the data: markers with a minor allele frequency > 5% and a call rate > 95% were kept, while non-informative monomorphic markers were discarded. For marker filtration, VCF tools V0.1.13 [48] software was utilized. The *dartR* package in R was used to calculate measures such as polymorphic information content (PIC), reproducibility, and call rate in order to examine the traits and distribution of the markers along the 16 sesame chromosomes [47]. The fraction of mutation types, including transversion (Tv) and transition (Ts), that are accountable for the observed polymorphism was also determined using the same package.

### 2.4. Genetic Relationship Visualization and Diversity Analysis

SNP marker information and genetic diversity analyses, including minor allele frequency parameter, were performed using TASSEL (v5.2.52) [49]. The adegenet package in R was also used to calculate observed and expected heterozygosity [50], while call rate and marker reproducibility were estimated using the dartR package in R [51]. Genetic structure was evaluated using a Bayesian model-based clustering approach implemented in STRUCTURE software (v2.3.4) [52]. Relationships among individuals were examined by computing a pairwise genetic distance matrix based on Euclidean distances in R. A NJ cluster was then constructed using the hclust function and exported in Newick format via the ape package for visualization and annotation in the Interactive Tree of Life (iTOL) platform (v6.5.2) (https://itol.embl.de/, accessed 12 December 2025) [53]. We emphasize that this analysis represents a genetic relationship visualization rather than a phylogenetic reconstruction, as it is applied to within-species diversity where reticulate events (hybridization and admixture) are expected.

### 2.5. Collection Diversity and Cluster Analysis

Filtered SNPs were used to investigate collection diversity within the germplasm using a Bayesian clustering approach implemented in STRUCTURE [54]. Binary files generated from the VCF data were further subjected to admixture analysis using the adegenet package in R [55]. The optimal number of genetic clusters (K) was determined through k-means clustering by testing k values ranging from 2 to 6, with competing solutions evaluated using the Bayesian Information Criterion (BIC) [56]. Based on admixture results, accessions with membership coefficients (q-values) ≥ 60% were assigned to specific groups, while those with Q-values < 60% were classified as admixed [57].

Principal component analysis (PCA) was performed in TASSEL v5.2.60, and scatter plots of sesame accessions were generated using the first two principal components (PC1 and PC2). Genetic diversity parameters, including private alleles, private SNPs, expected heterozygosity (He), and observed heterozygosity (Ho), were calculated for the identified subpopulations. To validate the model-based collection diversity inferred by STRUCTURE using a model-free approach, discriminant analysis of principal components (DAPC) was conducted with the adegenet package [58] in R v3.5.0 [50]. DAPC was applied to confirm the best-fitting number of clusters among the sesame accessions. This multivariate method combines sequential k-means clustering with model selection to infer and describe genetic structure, with the optimal K identified as the point beyond which further increases result in negligible changes in BIC values [59].

### 2.6. Analysis of Molecular Variance (AMOVA)

The genetic structure of the population was assessed using analysis of molecular variance (AMOVA) [60] implemented in the poppr package in R v2.2.4 [61,62]. Following the approach described by [63], AMOVA was applied to partition total genetic variation into components attributable to differences among populations and within populations.

## 3. Results

### 3.1. SNP Markes Summary

**Figure 2:**
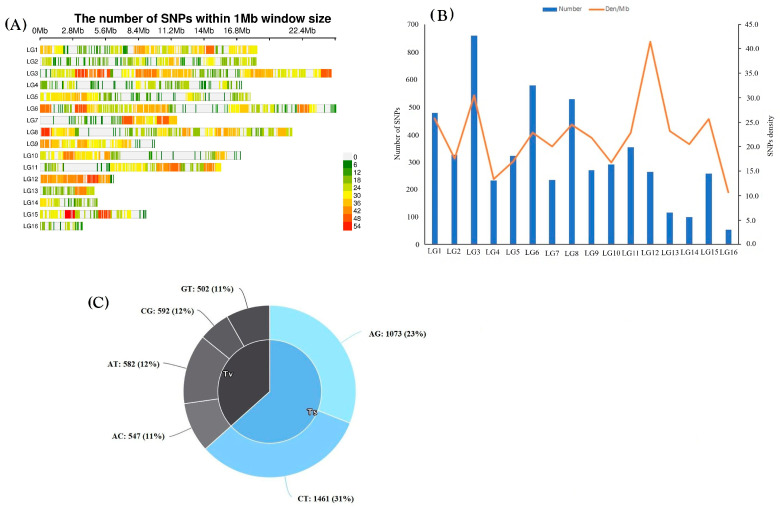
SNP marker summary. (**A**) Distribution of SNP markers within 1 Mb windows across sixteen linkage groups, (**B**) number and density of SNP markers in each linkage group, and (**C**) SNP mutation types identified among 5163 SNP markers used in analysis of sesame accessions.

In total, 12,302 SNP markers were initially obtained from the 188 sesame accessions. After applying filtering criteria, minor allele frequency (MAF) > 5% and missing data < 10%, a total of 7139 markers (58%) were removed. The remaining dataset was subsequently imputed. A final set of 5163 high-quality SNP markers (42%) met the required criteria and was retained for downstream genetic diversity analyses. All genome-wide SNPs were distributed across all 16 chromosomes but showed clear heterogeneity in density (Figure 2A). Using a 1 Mb sliding window, SNP density ranged from 0 to 54 SNPs/Mb, revealing heterogeneity in polymorphism levels across the genome. The total number of SNPs varied markedly across the sixteen chromosomes (Figure 2B). LG3, LG6, and LG8 contained the highest numbers of markers, each exceeding 500 SNPs, while LG13, LG14, and LG16 had the lowest counts (<150 SNPs). SNP density per megabase showed a slightly different trend, with LG12 exhibiting the highest density (>40 SNPs/Mb), despite having a moderate total SNP count. Moderate densities (20–30 SNPs/Mb) were recorded on LG1, LG3, LG6, LG8, LG11, and LG15, whereas LG7, LG13, LG14, and LG16 showed the lowest densities.

In the analyzed sesame genomes, transition-type SNPs were more frequent (54%) than transversion-type SNPs (46%), resulting in a transition-to-transversion (Ts/Tv) ratio of 1.17:1 (2788/2375) (Figure 2C). More A/T and C/G transitions were observed than G/T and A/C transitions. The frequency of the two transitions (A/G, C/T) was observed to be higher than the four transversions (A/C, G/T, C/G, and A/T), with C/T having the highest frequency of 31%, while the lowest frequency among the six allele combinations was A/C and G/T with 11%. The frequencies of the four transversion types were 11%, 11%, 12%, and 12% for A/C, G/T, C/G, and A/T, respectively (Figure 2C).

### 3.2. Genetic Relationship Visualization Analysis

**Figure 3:**
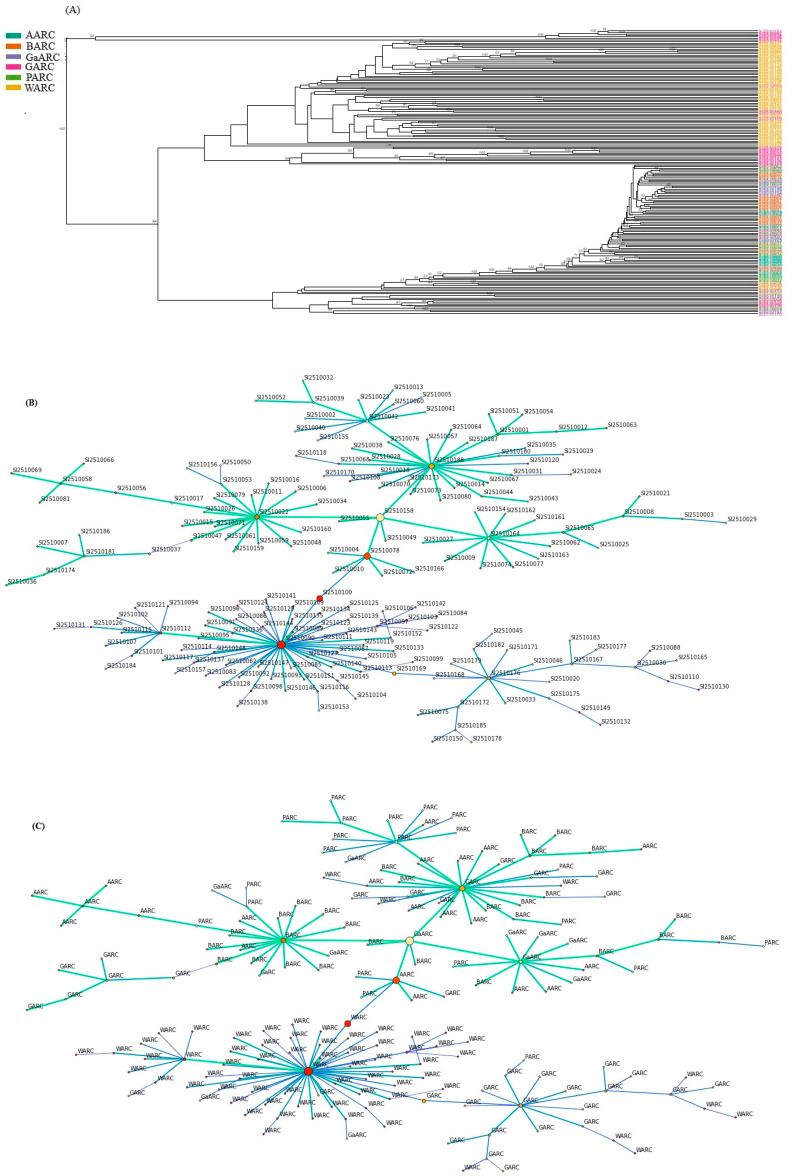
The cluster analysis using DArTseq-SNP markers for genetic relationship visualization among 188 sesame accessions. (**A**) Neighbor-Joining (NJ); (**B**) networking using accessions; and (**C**) networking using accessions sourced from institutes. The color indicate the source of centers.

Neighbor-Joining (NJ) tree-based visualizations revealed clear genetic relationships and differentiation among the 188 sesame accessions collected from the six research centers (AARC, BARC, GaARC, GARC, PARC, and WARC) (Figure 3A–C). The accessions clustered into three major genetic cluster groups, each further divided into multiple well-supported subgroups. This hierarchical clustering pattern indicates substantial genetic diversity within the panel. Although there was some degree of mixing among locations, several subclusters showed a strong tendency for samples from the same research center to group. For example, accessions from WARC and GARC formed several distinct and compact subclusters, suggesting closer genetic relatedness within these groups. In contrast, accessions from AARC and BARC were more widely dispersed across the tree, reflecting greater within-location diversity or shared ancestry with materials from other centers.

### 3.3. Genetic Diversity

The number of accessions, private alleles, private SNPs, allelic diversity, nucleotide diversity, heterozygosity, gene diversity, and fixation index across the research centers’ collections are shown in Table 1. The Werer Agricultural Research Center (WARC) collections showed higher heterozygosity (Ho = 0.292), and the GaARC collections showed higher gene diversity (He = 0.325) than other regions, while those from BARC and AARC had the lowest heterozygosity values (Ho = 0.131 and 0.138, respectively). The genetic diversity within each group/region revealed that the GaARC and PARC collection possess a comparable level of genetic diversity. The comparison of the total gene diversity analysis showed that the GaARC collection (He = 0.325) revealed the highest diversity, followed by the PARC (He = 0.282), BARC (He = 0.197), WARC (He = 0.185), GARC (He = 0.119), and AARC (He = 0.102) collections. A similar results trend was obtained using the fixation index across research centers‘ collections, in which the GaARC region had the highest value (Fst = 0.291), while the lowest was found for AARC (Fst = −0.123) (Table 1).

**Figure 4:**
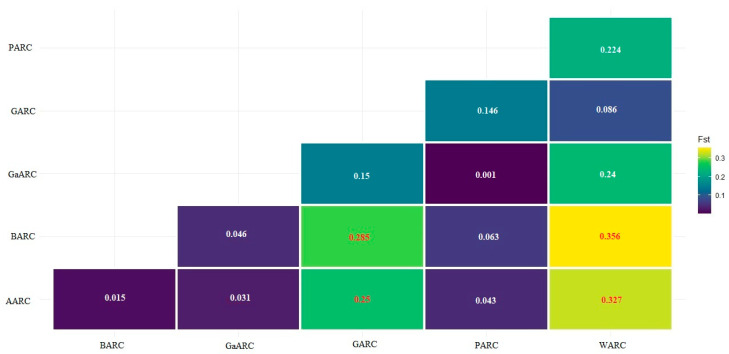
Institutional accession collection pairwise genetic differentiation index (FST) values of sesame research center collections (red number just for contrasting background for visibility of the numbers).

The pairwise genetic differentiation (FST) values revealed variable genetic structuring among sesame collections from different research centers, with low to moderate differentiation predominating, suggesting substantial gene flow or shared ancestry likely driven by germplasm exchange and the predominantly self-pollinating nature of sesame (Figure 4). Very low differentiation was observed between GaARC–PARC (Fst = 0.001) and BARC–AARC (0.015), as well as among AARC–GaARC (0.031), BARC–GaARC (0.046), and AARC–PARC (0.043), reflecting strong genetic similarity and overlap among these collections. In contrast, moderate differentiation was mainly associated with WARC; particularly for BARC–WARC (0.356) and AARC–WARC (0.327), the highest Fst values were observed, indicating greater genetic divergence that may reflect geographic isolation, distinct selection histories, or limited germplasm exchange. Moderate differentiation was also evident for GARC–BARC (0.285) and GARC–AARC (0.250) (Figure 4).

### 3.4. Principal Component Analysis (PCA)

**Figure 5:**
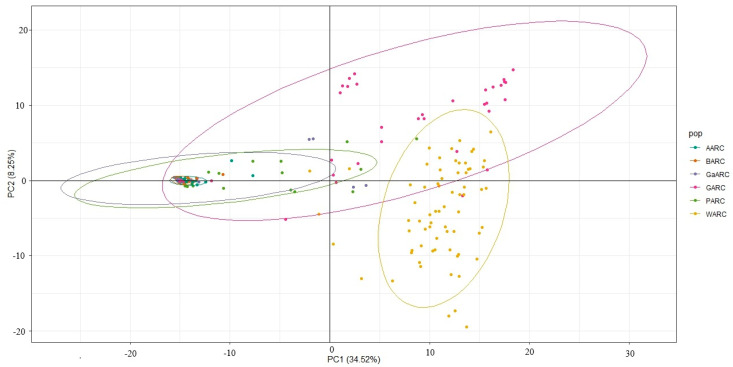
Principal component analysis showing the clustering between the geographical regions.

The principal component analysis (PCA) revealed clear genetic structuring among the sesame accessions collected from the six research centers (AARC, BARC, GaARC, GARC, PARC, and WARC) (Figure 5). PC1 (34.52%) and PC2 (8.25%) together explained 42.77% of the total genetic variation. Accessions from WARC formed a distinct and well-separated cluster along the positive axis of PC1, indicating substantial divergence from the other populations. GARC also showed a wide dispersion, forming a partially separated cluster mainly along PC2, suggesting high within-population variability. In contrast, accessions from AARC, BARC, GaARC, and PARC clustered closely near the origin, reflecting relatively lower genetic differentiation and higher similarity among these regions. The overall pattern highlights strong genetic divergence in WARC and GARC collections, while the remaining populations share more overlapping genetic backgrounds.

### 3.5. Discriminant Analysis of Principal Components (DAPC)

**Figure 6:**
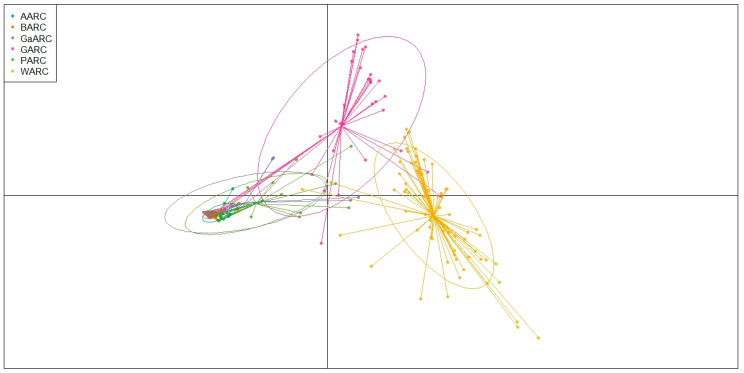
DAPC analysis showing the relationship between the geographic regions, with each color representing one cluster.

**Figure 7:**
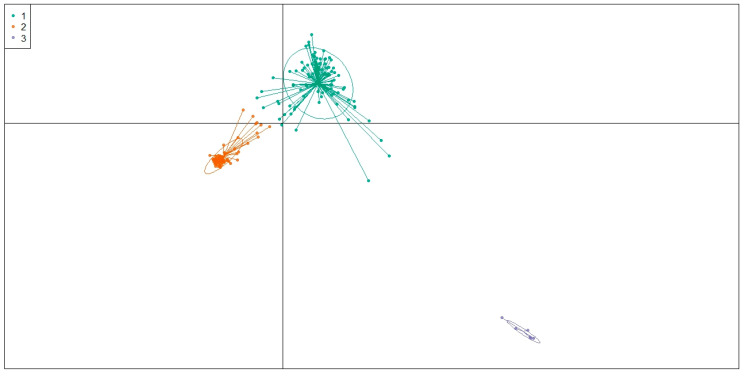
Genetic networks for all genetic groups, with node sizes indicating genetic relationships between different accessions.

The DAPC clearly separated the sesame accessions into distinct genetic clusters corresponding to their geographical origins (Figure 6). DAPC scatterplot and membership vectors show that WARC forms a well-defined and strongly differentiated cluster, indicating pronounced genetic divergence from the other accessions. GARC also separates clearly, with long membership vectors suggesting high within-population variability and strong discriminatory power of the discriminant functions. In contrast, accessions from AARC, BARC, GaARC, and PARC exhibit substantial overlap and compact clustering near the center, reflecting their relatively similar genetic backgrounds and low genetic cluster differentiation. Overall, the DAPC results confirm strong genetic structure driven mainly by the divergence of WARC and GARC collections, while the remaining four regions share highly overlapping genetic compositions. The DAPC, based on the optimal number of genetic clusters (K = 3), revealed a clear separation of the sesame accessions into three well-defined groups (Figure 7). Cluster 1 (green) formed a broad and widely dispersed group, indicating high within-cluster variability. Cluster 2 (orange) was tightly packed, suggesting relatively low internal diversity and strong genetic cohesion. Cluster 3 (purple) appeared as a small and completely isolated cluster, reflecting a highly distinct genetic lineage separated from the other two groups. The strong separation among the three clusters highlights pronounced genetic structuring within the entire collection and confirms the presence of at least three major genetically differentiated groups in the germplasm.

### 3.6. Collection Diversity Analysis

**Figure 8:**
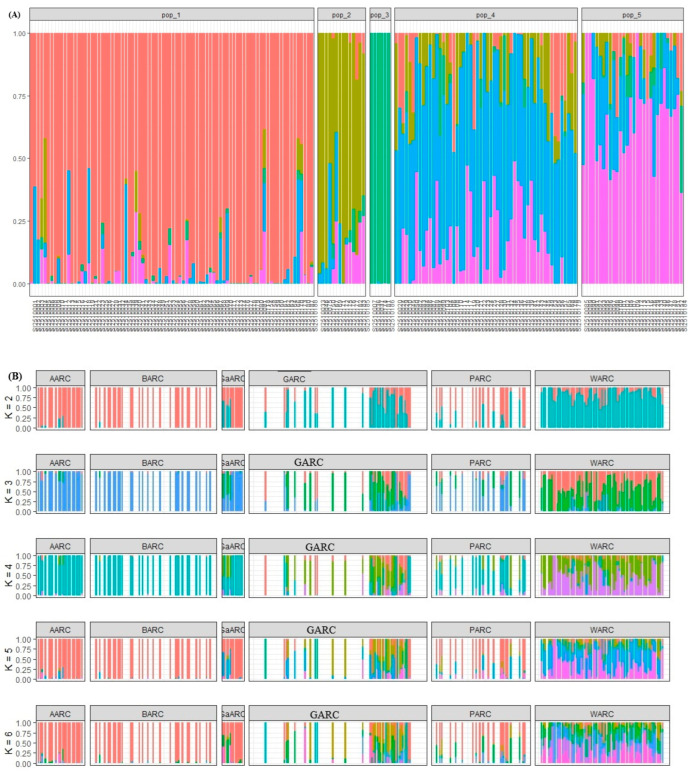
Collection diversity: (**A**) estimated collection diversity of sesame accessions assessed by STRUCTURE, in k = 5, where each color represents one cluster, and (**B**) collection diversity generated by the ADMIXTURE model among 188 sesame accessions (k = 2 top to k = 6 bottom). Each vertical bar represents an accession, partitioned into up to k colored segments.Red color Pop_1 group, light green represent Pop_2; green color represent Pop_3, light blue represent Pop_4 and Pink color represent Pop_5, and blue-green represent Pop_6.

The model-based Bayesian cluster analysis in STRUCTURE visualized the genetic structure of the research centers’ collection of sesame accessions. The STRUCTURE analysis (k = 5) grouped the 188 sesame accessions from AARC, BARC, GaARC, GARC, PARC, and WARC into five genetic clusters (Figure 8A). Three clusters (pop_1, pop_2, and pop_3) showed mostly pure ancestry, representing well-differentiated gene pools likely maintained within specific centers. In contrast, pop_4 and pop_5 displayed strong admixture, indicating extensive gene flow and germplasm exchange among centers, particularly those with active breeding and seed-sharing systems.

The multi-K STRUCTURE analysis (k = 2 to k = 6) showed clear differences in genetic composition and admixture levels among sesame accessions collected from the six research centers (Figure 8B). AARC and BARC form almost completely pure clusters, indicating genetically uniform and less-admixed gene pools (k = 2). GaARC and PARC show mixed ancestry, while WARC displays strong admixture, suggesting greater genetic heterogeneity and historical gene flow (k = 2; Table 2). AARC and BARC remain homogeneous, each dominated by a single genetic lineage (k = 3). GaARC and PARC begin to split into two or more subgroups, while WARC continues to show high admixture, reflecting a more diverse germplasm base (k = 3). AARC and BARC still cluster tightly with minimal substructure, while GaARC, GARC, and PARC show increasing subdivision, indicating the presence of multiple genetic backgrounds within these centers (K = 4). WARC again forms complex, highly admixed clusters, reinforcing its status as the most genetically diverse collection (k = 4). AARC and BARC retain predominantly single-ancestry profiles, reflecting conserved local breeding lines. GaARC and GARC show moderate admixture, indicating germplasm exchange or shared ancestry with neighboring centers (k = 5). PARC and especially WARC exhibit strong multi-ancestry patterns, confirming broad genetic mixing (k = 5). The overall pattern stabilizes, AARC and BARC remain genetically distinct with very low admixture (k= 6). GaARC, GARC, and PARC show intermediate levels of structure and admixture, forming several subgroups (k = 6). WARC consistently displays the highest admixture, representing the most diverse and genetically mixed gene pool (k = 6). Across all K values, AARC and BARC maintain highly homogeneous and distinct genetic clusters, while GaARC, GARC, and PARC show moderate admixture and substructures. WARC consistently exhibits the highest admixture and genetic diversity, indicating extensive germplasm mixing and a broad ancestral base.

### 3.7. Molecular Variance (AMOVA)

The analysis of molecular variance (AMOVA) showed that, when accessions were grouped by research center collections, 70% of the total genetic variation was distributed within accession groups, while 30% occurred among groups (Table 3). The among-group differentiation was significant, with a PhiPT value of 0.298 (*p* = 0.001), suggesting measurable accession groups structuring but with considerable gene flow or shared ancestry among institutional collections. When grouped based on collection diversity, AMOVA revealed a higher proportion of variation among accession groups (43%), with 57% of the variation within groups (Supplementary Table S3). This is used to quantify the magnitude of differentiation already identified by the clustering algorithms, rather than as independent proof of biological truth. This grouping also showed significant genetic differentiation, with a PhiPT value of 0.429 (*p* = 0.001), indicating stronger genetic differentiation under structure-based grouping compared to institutional collections. This demonstrates that genetic structure explains genetic differentiation better than sampling location, reflecting underlying genetic subdivisions not strictly aligned with geography.

## 4. Discussion

Sesame is a predominantly self-pollinating oilseed crop, though occasional outcrossing occurs and is exploited in breeding programs. Predominant selfing leads to low heterozygosity, high homozygosity within accessions, strong linkage disequilibrium, and pronounced differentiation among accessions [64,65]. Consequently, elevated *FST* values observed in this study are interpreted primarily as reflecting restricted effective recombination and fixation within accession groups, rather than ongoing gene flow among research center collections. Occasional outcrossing, combined with historical germplasm exchange and regeneration practices, introduces admixture and contributes to within-accession groups [66]. Overall, the observed patterns of uniformity, admixture, and divergence are shaped by selfing, rare hybridization, and ex situ management, providing essential context for interpreting heterozygosity, differentiation metrics (including *FST*), and clustering in these managed germplasm collections.

### 4.1. SNP Markers Density and Genome-Wide Variation Patterns

The high filtering rate, where 58% of raw SNP markers were removed, is consistent with the findings of [67], yielding 5163 high-quality SNPs (42%), and aligns with previous sesame studies using similar genotyping-by-sequencing approaches, which typically retain 40–55% of raw markers after stringent quality control [68]. The observed deficit of heterozygotes (mean He = 0.201 > Ho = 0.193) is an indication of self-pollination and has been consistently documented across diverse sesame germplasm panels [69,70,71]. In line with earlier findings of heterogeneous SNP density in sesame [68,70], genome-wide SNPs were unevenly distributed across the 16 chromosomes, with hotspots on LG6, LG8, LG12, and LG15, and extended low-polymorphism regions on LG4, LG7, LG10, and LG13. While shorter chromosomes (LG9–LG16) had fewer markers, the longest chromosomes (LG1–LG8) typically had more SNPs; however, LG12 showed the highest density per megabase despite moderate total SNP counts. This pattern provides adequate genomic resolution for downstream diversity and association analyses. Furthermore, the predominance of transition mutations (Ts/Tv = 1.17:1), particularly C/T transitions (31%), mirrors the known mutational bias in plants due to spontaneous cytosine deamination and matches spectra from recent resequencing studies [72]. This consistency in the mutation spectrum underscores the biological validity of the filtered SNP set. The heterozygosity patterns, mutation spectrum, and uneven SNP distribution taken together show that the dataset is reliable for thorough genomic analyses and captures the inherent genomic variation of sesame.

### 4.2. Unequal Distribution of Genetic Diversity of Sesame Accessions Across Ethiopian Research Centers

Our findings reveal significant heterogeneity in genetic variation and clear accession stratification, offering valuable insights for conservation and breeding strategies. The model-based clustering and NJ cluster analysis confirmed substantial genetic differentiation among accessions from the six centers, forming three major genetic cluster groups. While the tree provides a clear hierarchical view of the collection’s structure, it is a simplified representation of the underlying genetic relationships. Given the history of breeding and germplasm exchange in these research centers, some degree of reticulated ancestry is likely, which may not be fully captured by a strictly bifurcating model. This hierarchical genetic structure is consistent with patterns observed in other crops, where geographic and institutional isolation can drive divergence [73]. The tendency for accessions from specific centers (notably, GaARC WARC and GARC) to form distinct, compact subclusters indicates strong genetic identity and potentially limited gene flow with external sources. In contrast, the wider dispersion of AARC, PARC, and BARC accessions across the tree suggests either a historically diverse founding population or more extensive exchange and introgression with materials from other centers, use of similar breeding materials, or localized adaptation, a phenomenon documented in sesame collections in China and India [74,75]. The presence of mixed-center clusters further suggests historical gene flow and sharing of germplasm among research centers, contributing to the moderate genetic differentiation observed. From a conservation perspective, the distinct clustering and unique lineages associated with GaARC, WARC, and GARC highlight these collections as priority targets for both in situ and ex situ conservation, as they harbor rare alleles and divergent accessions that may be lost if not properly maintained. For genetic improvement, the clear genetic divergence among clusters provides valuable opportunities for parental selection, as crosses between genetically distant accessions, particularly those from GaARC or WARC and more uniform populations, could enhance heterosis and broaden the genetic base of Ethiopian sesame breeding programs.

### 4.3. Genetic Diversity Metrics Among Research Center-Based Sesame Accessions

Our analyses reveal a structured yet interconnected landscape of genetic diversity among sesame accessions maintained across Ethiopian research centers. Diversity metrics identified GaARC as a major reservoir of variation, exhibiting the highest gene diversity (He = 0.325) and fixation index (Fst = 0.291) (Table 1), along with numerous private alleles, indicating unique genetic resources valuable for breeding and adaptation. PARC also showed high allelic and nucleotide diversity, emphasizing its importance for broadening the breeding pool. WARC displayed comparatively high heterozygosity (Ho = 0.292) and moderate-to-high differentiation from other centers, reflecting both substantial internal variation and genetic distinctiveness. In contrast, AARC and BARC consistently exhibited lower heterozygosity and gene diversity (Ho = 0.131–0.138, He = 0.102–0.197) (Table 1), suggesting a narrower genetic base potentially shaped by historical selection, founder effects, or genetic erosion, which may increase vulnerability to environmental stresses [76].

Consistent with these patterns, pairwise differentiation estimates indicated that most collections are weakly differentiated, with very low *FST* values among AARC, BARC, GaARC, and PARC (e.g., GaARC–PARC = 0.001; BARC–AARC = 0.015) (Figure 4), reflecting shared germplasm sources, frequent seed exchange, and comparable breeding histories [13]. In contrast, WARC—and to a lesser extent GARC—showed moderate differentiation (e.g., WARC–BARC = 0.356; WARC–AARC = 0.327; GARC–BARC = 0.285; GARC–AARC = 0.250) (Figure 4), highlighting their potential as sources of novel alleles for traits such as drought tolerance and disease resistance [77].

Population structure analyses supported these observations. STRUCTURE analysis (optimal K = 5) partitioned the 188 accessions into five genetic clusters, ranging from relatively homogeneous clusters with limited admixture to highly admixed groups. Principal component analysis separated more divergent collections, such as WARC, from a core cluster of genetically similar centers (AARC, BARC, GaARC, PARC), while Neighbor-Joining tree visualization highlighted both highly similar clusters among core centers and distinct lineages in more differentiated collections. Clusters with predominantly pure ancestry indicate the maintenance of locally adapted gene pools with restricted gene flow, whereas the extensive admixture observed—particularly in WARC—reflects its historical role as an active germplasm hub engaged in systematic collection and exchange [75,78,79].

The AMOVA results further reinforced these patterns. Moderate but significant differentiation among collection sites (PhiPT = 0.298, *p* = 0.001) (Table 3), with 70% of the total variation residing within populations, aligns with the partial overlap of accessions from different centers observed in NJ clustering. This overlap likely reflects shared ancestry and seed exchange among institutes, a pattern commonly observed in self-pollinated crops with regional germplasm movement [80,81]. When accessions were grouped according to inferred genetic clusters, the proportion of variation among clusters increased to 43% (PhiPT = 0.429, *p* = 0.001) (Table S3), indicating stronger differentiation and confirming the distinct groups identified in STRUCTURE analyses with reduced admixture. These results demonstrate that genetic differentiation in this panel is better explained by underlying genomic structure than by geographic origin alone, consistent with previous diversity and GWAS panels [59].

Overall, the concordance among diversity metrics, pairwise differentiation, NJ, STRUCTURE, and AMOVA reveals a robust and hierarchical genetic structure. Most Ethiopian sesame collections are genetically interconnected, reflecting shared germplasm and seed exchange, while WARC and, to a lesser extent, GARC represent more differentiated gene pools harboring unique alleles. This structure, characterized by substantial within-group diversity alongside distinct genetic clusters, provides a strong foundation for association mapping, parent selection, and breeding programs, while ensuring the long-term sustainability of sesame improvement efforts [80,82].

The clear genetic divergence of WARC, as a distinct cluster in PCA space, identifies it as a critical reservoir of diversity. Its broad ancestral base likely results from the amalgamation of materials from multiple origins. Conversely, the tight, homogeneous clustering of AARC and BARC accessions near the PCA origin, alongside their pure ancestry, suggests that these centers maintain genetically uniform breeding lines or landraces. While such purity is valuable for preserving specific traits, it also indicates a narrow genetic base, which can limit adaptive potential and is a common concern in structured breeding programs [76]. The intermediate and substructured nature of GaARC, GARC, and PARC reflects a more moderate history of exchange. These centers show evidence of both distinct subgroups and admixture, suggesting they possess internal diversity while also sharing ancestry with neighboring pools. The minimal differentiation among AARC, BARC, GaARC, and PARC in the PCA supports this interpretation of shared genetic background within a core group, a pattern similarly observed in regional sesame populations in South Asia [74]. The wide dispersion of GARC along PC2 further highlights significant within-population variation, another key component of overall diversity.

The Discriminant Analysis of Principal Components (DAPC) provides robust, complementary evidence of pronounced genetic structure within the sesame germplasm collection, effectively discriminating among populations based on their geographic and institutional origins. The separation of accessions into distinct clusters validates and refines the patterns identified by PCA, highlighting specific centers as drivers of major genetic divergence. The pronounced isolation of the WARC population in DAPC space, forming a well-defined and distant cluster, underscores its status as a highly divergent genetic pool. This strong differentiation is consistent with findings in other crops where specific germplasm hubs, often located in regions of secondary diversity or characterized by intensive historical collection efforts, accumulate and preserve unique alleles, leading to significant genetic distinctness [13]. Similarly, the separation and long membership vectors of the GARC population indicate not only differentiation from other groups but also considerable within-population variability. This pattern suggests that GARC’s germplasm may comprise several sub-lineages or admixed individuals, indicating collections that have incorporated materials from diverse sources, thereby increasing their value for breeding [83].

In contrast, the substantial overlap and compact clustering of accessions from AARC, BARC, GaARC, and PARC near the DAPC centroid reflect a shared genetic background and low inter-population differentiation. This genetic homogeneity among centers is indicative of either common ancestral origins, historical seed exchange within a regional network, or parallel selection for similar adaptive traits, a phenomenon frequently observed in adjacent agricultural zones [79]. The DAPC result confirming K = 3 as an optimal clustering level reveals a simplified but meaningful hierarchy: one broad, variable group (Cluster 1), one cohesive, uniform group (Cluster 2), and a small, isolated lineage (Cluster 3). This tripartite structure suggests that, beyond the specific institutional origins, the entire collection is stratified into major gene pools representing (1) a diverse, admixed background, (2) a conserved, pure lineage, and (3) a unique genetic resource. The tight, isolated nature of Cluster 3 is particularly noteworthy. Such distinct, small clusters often represent rare landraces, breeding lines with unique ancestries, or relics of older varietal groups. Their preservation is critical, as they may harbor novel alleles for stress tolerance or quality traits that have been lost from mainstream breeding pools, a concern raised in assessments of genetic erosion in crops like sesame and rice [84]. Conversely, the broad dispersion of Cluster 1 aligns with the high-admixture profiles seen in WARC and parts of GARC, reinforcing their role as repositories of genetic variance. In conclusion, the DAPC analysis definitively captures the major axes of genetic differentiation within the collection, identifying WARC and GARC as key divergent populations and revealing a core group of genetically similar centers. The resolution into three primary genetic clusters provides a practical and powerful lens through which to manage, conserve, and utilize this germplasm. By aligning breeding strategies with this inherent genetic architecture, we can more efficiently harness the full spectrum of diversity to enhance the resilience and productivity of sesame.

## 5. Conclusions

This study provides a comprehensive genome-wide assessment of genetic diversity and population structure in 188 Ethiopian sesame accessions using high-quality DArTSeq-based SNP markers. Four seedlings per accession were sampled to ensure successful DNA extraction and to represent each accession, rather than to quantify within-accession genetic variation. Consequently, the analyses were designed to infer broader patterns of genetic diversity and structure among accessions and ex situ collections rather than fine-scale intra-accession variation. The large number of informative SNPs distributed across all sixteen chromosomes revealed substantial genetic variation and a heterogeneous genomic landscape, confirming the robustness of the dataset for genetic analysis of accession groups. Overall levels of expected heterozygosity exceeded observed heterozygosity, reflecting the predominantly self-pollinating nature of sesame, while the prevalence of transition-type mutations and uneven SNP density further highlighted the intrinsic genomic characteristics of the crop.

Consistent results from STRUCTURE, PCA, DAPC, and NJ cluster analysis, pairwise FST, and AMOVA demonstrated a clear but complex population structure shaped by both historical gene flow and institutional germplasm management. Certain research centers, particularly WARC and GARC, emerged as highly diverse and genetically distinct reservoirs, whereas AARC and BARC showed relatively homogeneous and narrowly based gene pools. AARC and BARC appear highly homogeneous with predominantly pure ancestry across multiple K values in STRUCTURE, whereas the same collections are widely dispersed in the neighbor-joining (NJ) tree. This difference reflects methodological contrasts rather than a true inconsistency. STRUCTURE emphasizes shared ancestry and between-cluster differentiation, which may obscure fine-scale variation within collections, while the NJ tree is based on genetic distances and is sensitive to subtle variation and admixture. Consequently, the dispersion of AARC and BARC accessions in the NJ tree reflects intra-collection diversity, whereas STRUCTURE highlights their shared genetic background, rendering the two approaches complementary. The AMOVA results confirmed that most genetic variation resides within populations, yet structure-based grouping explained a greater proportion of among-population variance than geographic origin alone, underscoring the importance of underlying genomic structure over simple spatial classification.

Moreover, the genetic diversity and structure of sesame collections across Ethiopian research centers revealed extensive germplasm exchange and shared curation histories, as indicated by weak genetic structure and significant genetic overlap among centers. These patterns show that institutional origin alone does not define separate genetic pools, highlighting the need for diversity-based organization of collections. The observed moderately differentiated and highly admixed groups provide a solid foundation for developing compact core sets that maximize allelic diversity while minimizing redundancy, facilitating efficient parental selection and pre-breeding. Collectively, these findings demonstrate the strategic value of divergent and admixed populations for crop improvement, supporting efficient germplasm conservation and serving as a foundation for designing structured parental panels specifically for use in association mapping or the development of multi-parent populations. Integrating this genomic information with multi-environment phenotypic data will enable the identification of trait-associated loci and accelerate marker-assisted and genomic selection for yield stability, drought tolerance, and other economically important traits, thereby enhancing sesame breeding programs and strengthening long-term food and economic security.

## Figures and Tables

**Table 1:** Genetic parameter estimates based on 5163 SNPs among sesame subpopulations.

Populations	Genetic Parameters							
	Ng	Pa	Ps	Ad	Nd	Ho′	He′	Fst
Based on Research Center Collections								
AARC	21	0	0	1.404	0.2048	0.131	0.102	−0.123
BARC	28	0	0	1.743	0.3921	0.166	0.197	0.138
GaARC	12	113	20	1.930	0.6477	0.223	0.325	0.291
GARC	35	6	1	1.480	0.2385	0.138	0.119	−0.066
PARC	21	152	24	1.882	0.5644	0.292	0.282	0.002
WARC	71	0	0	1.741	0.3726	0.207	0.185	−0.065
Mean		45.2	7.5	1.7	0.4	0.2	0.2	0.03

**Table 2:** Proportion of membership of each predefined accession group in each of clusters obtained at best k (k = 5).

Research Center Collections	Number of Accessions	Admixed Individual	Proportion of Membership in Each Cluster (%)				
			Cluster I	Cluster II	Cluster III	Cluster IV	Cluster V
**STRUCTURE**							
AARC	21	0	21	0	0	0	0
BARC	28	2	26	0	0	0	0
GaARC	12	2	8	0	0	2	0
GARC	36	13	5	11	3	4	0
PARC	21	7	14	0	0	0	0
WARC	70	35	0	0	0	17	19
	188	31%	39%	6%	2%	12%	10%

**Table 3:** Analysis of molecular variance among and within sesame accession groups.

Source of Variation	Df	SS	MS	EV	PV	Statistics			*p*-Value
						PhiPT	PhiPT Max	Phi’PT	
Among accession groups	5	46,887.540	9377.508	298.304	30%				
Within accession groups	182	127,753.864	701.944	701.944	70%				
Total	187	174,641.404		1000.248	100%	0.298	0.730	0.409	0.001

## Data Availability

The original contributions presented in this study are included in the article. Further inquiries can be directed to the corresponding author.

## Supplementary Materials

The following supporting information can be downloaded at: https://www.mdpi.com/article/10.3390/genes17030300/s1, Table S1: List of sesame accessions with their collection research centers, Table S2: Genetic diversity parameters across chromosomes, and Table S3: Analysis of molecular variance among and within sesame accession groups according to the K-group.

## Abbreviations

The following abbreviations are used in this manuscript:

AARC	Assosa Agricultural Research Center
BARC	Bako Agricultural Research Center
GaARC	Gambella Agricultural Research Center
GARC	Gondar Agricultural Research Center
PARC	Pawe Agricultural Research Center
WARC	Werer Agricultural Research Center
Ho	Observed heterozygosity
He	Expected heterozygosity
Ht	Overall gene diversity
Dst	Gene diversity among samples
Dst	Locus-Specific Differentiation
Fis	Inbreeding Coefficient
H′	Shannon diversity index
Ng	Number of genotypes
Pa	Private alleles
Ps	Private SNPs
Ad	Allelic diversity
Nd	Nucleotide diversity
He′	Gene diversity
Ho′	Heterozygosity
Fst	Fixation index across the population
Df	Degrees of freedom
SS	Sum of squares
MS	Mean square
EV	Estimated variance
PV	Percentage variance
PhiPT	Genetic differentiation among populations
Phi’PT	Standardized genetic differentiation
PhiPT max	Maximum possible population differentiation
