# Supplementary material for: Genetic Diversity and Collection Structure Studies of Sesame (Sesamum indicum L.) Accessions Across Ethiopian Research Centers
Source: Genes (Basel). 2026 Feb 28;17(3):300. doi: 10.3390/genes17030300 (PMC13026398; doi:10.3390/genes17030300)
Supplement: Supplementary file 1 [file genes-17-00300-s001.zip › genes-4146220-supplementary.pdf]

Supplementary Table S1: List of sesame genotypes with their collection research centers.

No.	Genotype	Collection Site	No.	Genotype	Collection Site	No.	Genotype	Collection Site
1	Yale	BARC	64	AsARC-acc-3	AARC	127	Gobyo-82	WARC
2	EBI 9021	PARC	65	Chalasa	BARC	128	Acc NS 002 Mhmsl	WARC
3	Hachalu	BARC	66	AsARC-acc-S-006	AARC	129	NN-0089 (2)	WARC
4	Gonder-F-123/10	PARC	67	24290	BARC	130	Setit Standard Check	WARC
5	HUMERA-1-sel-1	PARC	68	AsARC-acc-S-10	AARC	131	Acc-205-191	WARC
6	9692	BARC	69	AsARC-acc-SA-007	AARC	132	WARC-60/Different	WARC
7	P# 87	GARC	70	AsARC-acc-SA-011	AARC	133	NN-0064-LTMS	WARC
8	Dicho x EW006-1-1-1	BARC	71	AsARC-acc-S-001	AARC	134	Acc 00028	WARC
9	Walin	BARC	72	Ass-acc-71	AARC	135	Hirhir Kibebew eamm Sel-1	WARC
10	EBI 237994	PARC	73	Ass-acc-11	AARC	136	Bowja-Gobate Sel-3	WARC
11	BKC-091	BARC	74	Gida Ayana	AARC	137	Gobyo-83	WARC
12	17479	BARC	75	P#10	GARC	138	NN-0048	WARC
13	warc-068-gonderlet set	PARC	76	IBC-acc-17703	AARC	139	NN 0029 (2)	WARC
14	9025	BARC	77	Ass-acc-35	AARC	140	Kenya	WARC
15	9693	BARC	78	IBC-acc-202514	AARC	141	Unknown baja Sel-7-Tm1	WARC
16	17713	BARC	79	IBC-acc-237994	AARC	142	Tejereb Kokat Sel-3	WARC
17	EBI 25839	PARC	80	Ass-acc-105	AARC	143	Maru Sel-1	WARC
18	P# 224	GARC	81	AsARC-acc-SA-022	AARC	144	Acc-111-848	WARC
19	P# 76	GARC	82	WW-118	WARC	145	NN-027	WARC
20	P# 273	GARC	83	Hirhir Kibebew-hair-iless Se1-1	WARC	146	Ab x T-81 Sel-1	WARC
21	BG006 X EW023(2)-10-1-1	BARC	84	P#5	GARC	147	Bounia-firwuhe Sel-4	WARC
22	Bk-005	BARC	85	Fincha-22 (90)	WARC	148	Tejareb Giras	WARC
23	Adi-sel-2099	PARC	86	Hirhir	WARC	149	Hirhir Kibebew	WARC
24	P# 290	GARC	87	Acc 024-Sel-3	WARC	150	Acc-No. 019	WARC
25	WARC-068	PARC	88	Acc WW-001 (7)	WARC	151	Acc-203-616 Mumsi	WARC
26	BK-088-1	BARC	89	NN-0183-3 Mhms1	WARC	152	BCS-023	WARC
27	EBI 29071	PARC	90	Acc-202-889	WARC	153	GM-1	GaARC
28	EW003(1) x EW002-4-1-1	BARC	91	Acc-051-02-Sel 5(1)	WARC	154	GM-2	GaARC
29	WARC-082	PARC	92	Hirhir Kibebew early Sel-1	WARC	155	GM-3	GaARC
30	P# 67	GARC	93	Tejareb girar	WARC	156	GM-4	GaARC
31	17693	BARC	94	EW-020 (1)	WARC	157	GM-5	GaARC
32	EBI 9020	PARC	95	Abasena	WARC	158	GM-6	GaARC
33	P# 295	GARC	96	Acc-203-336 SPS	WARC	159	GM-7	GaARC
34	Dicho x Wama-11-1-1	BARC	97	Acc-00024 (1)	WARC	160	GM-8	GaARC
35	EBI 29396	PARC	98	VBSM-1026	WARC	161	GM-19	GaARC
36	P#227	GARC	99	Mexico 8	WARC	162	GM-10	GaARC
37	P#162	GARC	100	Acc-EW-008 (1)	WARC	163	GM-11	GaARC

38	9689	BARC	101	Acc-205-43	WARC	164	GM-12	GaARC
39	Benshangul-1	PARC	102	ET #17-03	WARC	165	P# 54	GARC
40	WARC-092	PARC	103	No. 458-1	WARC	166	P# 282	GARC
41	Gida Ayana	PARC	104	Gojam Azene Yohanis Sel-1	WARC	167	P# 203	GARC
42	EBI 208752	PARC	105	NN-0104	WARC	168	P# 252	GARC
43	EBI 27419	PARC	106	Acc-WW-001 (1)	WARC	169	P# 177	GARC
44	17712	BARC	107	Local-202	WARC	170	P# 238	GARC
45	EBI 208752	PARC	108	Acc-EW-08 (1)	WARC	171	P# 220	GARC
46	P# 13	GARC	109	EW-017 (1)	WARC	172	P# 104	GARC
47	BK-086-1	BARC	110	Setit	WARC	173	P# 217	GARC
48	17708	BARC	111	UCR-82-14 (N) 209	WARC	174	P# 145	GARC
49	19040	BARC	112	Acc-00021 Mhm-52	WARC	175	P# 78	GARC
50	WARC-070	PARC	113	Acc-203-616	WARC	176	P# 132	GARC
51	Hagalo	BARC	114	Hirhir baker Sel-4	WARC	177	P# 211	GARC
52	Abasena	PARC	115	Acc-203-3364	WARC	178	P# 188	GARC
53	EBI 28314	PARC	116	Bering bawang	WARC	179	P# 164	GARC
54	Dicho	BARC	117	Ab x T-85 Sel-3	WARC	180	P# 49	GARC
55	9019	BARC	118	Acc EW-020 (2)	WARC	181	P# 257	GARC
56	AsARC-acc-SA-017	AARC	119	BouNTG-2	WARC	182	P# 265	GARC
57	Ass-acc-17	AARC	120	Acc EW 010 (2)	WARC	183	P# 14	GARC
58	AsARC-acc-SA-020	AARC	121	Hirhir Muse Pump	WARC	184	P# 222	GARC
59	9028	BARC	122	Mexico-9	WARC	185	P# 119	GARC
60	IBC-acc-208752	AARC	123	T-85 x PARM-Sel-1 (1-1)	WARC	186	P# 246	GARC
61	BKC-057	BARC	124	Bounja-Gobate Sel-13	WARC	187	P# 249	GARC
62	AsARC-acc-85	AARC	125	Acc-031-Sel-1-14	WARC	188	P# 232	GARC
63	AsARC-acc-48	AARC	126	Sepnit Sel-6 Mhm 53	WARC			

Supplementary Table S2: Genetic diversity parameters across chromosomes.

Chr	Ho	He	Ht	Dst	Htp	Dstp	Fst	Fstp	Fis	Dest	MaF	MAF
LG1	0.185	0.201	0.237	0.036	0.244	0.043	0.115	0.132	0.151	0.059	0.81	0.19
LG2	0.146	0.161	0.192	0.031	0.198	0.037	0.111	0.127	0.170	0.049	0.843	0.157
LG3	0.180	0.191	0.232	0.040	0.240	0.049	0.130	0.148	0.132	0.065	0.811	0.189
LG4	0.186	0.197	0.236	0.039	0.243	0.046	0.114	0.131	0.110	0.065	0.803	0.197
LG5	0.205	0.208	0.242	0.034	0.249	0.041	0.110	0.128	0.075	0.058	0.81	0.19
LG6	0.199	0.213	0.259	0.046	0.268	0.055	0.144	0.165	0.126	0.075	0.781	0.219
LG7	0.191	0.199	0.237	0.037	0.244	0.045	0.120	0.138	0.094	0.062	0.815	0.185
LG8	0.197	0.206	0.252	0.047	0.262	0.056	0.147	0.168	0.112	0.076	0.787	0.213

LG9	0.229	0.232	0.277	0.045	0.286	0.054	0.129	0.148	0.060	0.076	0.78	0.22
LG10	0.204	0.205	0.237	0.032	0.243	0.038	0.106	0.122	0.093	0.054	0.808	0.192
LG11	0.218	0.221	0.273	0.052	0.283	0.062	0.151	0.171	0.086	0.084	0.772	0.228
LG12	0.208	0.209	0.247	0.038	0.255	0.046	0.118	0.136	0.094	0.065	0.793	0.207
LG13	0.246	0.252	0.292	0.040	0.300	0.048	0.117	0.134	0.076	0.068	0.758	0.242
LG14	0.133	0.148	0.173	0.025	0.178	0.030	0.091	0.105	0.110	0.040	0.86	0.14
LG15	0.181	0.196	0.248	0.052	0.259	0.063	0.164	0.187	0.147	0.085	0.779	0.221
LG16	0.178	0.184	0.252	0.067	0.265	0.081	0.203	0.229	0.044	0.106	0.761	0.239
Mean	0.193	0.201	0.243	0.041	0.251	0.050	0.129	0.148	0.105	0.068	0.798	0.202

Supplementary Table S3: Analysis of molecular variance among and within sesame accession groups according to the K-group.

Source of variation	Df	SS	MS	EV	PV	Statistics			P-value
						PhiPT	PhiPT max	Phi'PT	
Among accession groups	4	62051.187	15512.797	462.168	43%				
Within accession groups	183	112589.292	615.242	615.242	57%				
Total	187	174640.479		1077.410	100%	0.429	0.760	0.565	0.001
